# Supplementary material for: Initial state of DNA-Dye complex sets the stage for protein induced fluorescence modulation
Source: Nat Commun. 2019 May 8;10:2104. doi: 10.1038/s41467-019-10137-9 (PMC6506533; doi:10.1038/s41467-019-10137-9)
Supplement: Supplementary file 1 — Supplementary Informartion [file 41467_2019_10137_MOESM1_ESM.pdf]

Supplementary Information:

Initial state of DNA-Dye complex sets the stage for protein induced fluorescence modulation

Fahad Rashid[1], Vlad-Stefan Raducanu[1], Manal S. Zaher[1], Muhammad Tehseen[1], Satoshi Habuchi[1], and Samir M. Hamdan[1].

[1] King Abdullah University of Science and Technology, Division of Biological and Environmental Sciences and Engineering, Thuwal 23955, Saudi Arabia.

These authors contributed equally: Fahad Rashid, Vlad-Stefan Raducanu, Manal S. Zaher

Correspondence and requests for materials should be addressed to S.M.H. (email: samir.hamdan@kaust.edu.sa)

Supplementary Methods

Steady State Fluorescence

Steady state fluorescence measurements for FEN1/DF system were conducted at room temperature using Fluoromax-4 (HORIBA JOBIN YVON). The fluorescence intensities of DF substrates (DF-2,1 to DF-18,1) harboring pCy3 at the tip of their 5' flaps were measured in FEN1 reaction buffer (50 mM HEPES-KOH pH 7.5, 100 mM KCl, 5% (v/v) glycerol, 10 mM $CaCl_2$ and 1 mM DTT). For measurements in the presence of FEN1, a saturating concentration of FEN1 was added to the mixture of labeled substrates in FEN1 reaction buffer and allowed to reach equilibrium state after 3 mins incubation at room temperature. In both cases, pCy3 was excited at 535 nm ($\lambda_{max\text{-}ex}$) and emission spectra were collected between 550 and 700 nm. Both excitation and emission slit widths were set to 5 nm. Measurements were recorded with an integration time of 0.1 s. The emission spectra were corrected by subtracting the background emission of a blank solution comprised of FEN1 reaction buffer. For each measurement, the steady state fluorescence intensity was evaluated by integrating the corresponding emission spectrum with ± 5 nm (slit width) bounds around 565 nm ($\lambda_{max\text{-}em}$). The steady state (SS) fluorescence change upon FEN1 addition to the DF substrates was calculated as a percentage of the difference between the initial fluorescence intensity (I_i) of DF substrates and the final fluorescence intensity (I_f) after the addition of FEN1 to the initial fluorescence intensity (SS Fluorescence Change = 100 * (I_i -I_f)/ I_i). The reported measurements are the average of 3 independent replicates.

For oligo/RPA system, steady state fluorescence measurements of pCy3-labeled oligos were performed at room temperature using a microplate spectrofluorometer (TECAN infinite M1000) in RPA reaction buffer (50 mM HEPES-KOH pH=7.5, 50 mM KCl, 5% glycerol and 1 mM $MgCl_2$) in the absence and presence of RPA at saturating concentration. The samples were excited at 535 nm ($\lambda_{max\text{-}ex}$ of Cy3) and emission was collected at 565 nm ($\lambda_{max\text{-}em}$ of Cy3) with 5 nm slit width for both excitation and emission and an integration time of 0.1 s. The measurements were corrected by subtracting the emission of RPA reaction buffer used as a blank. The fluorescence change (%) upon addition of RPA was evaluated as described above.

Absorbance Measurements

The absorbance of pCy3-labeled DF substrates in the absence and presence of FEN1 was measured at room temperature using Thermo-Scientific™ Evolution™ Spectrophotometer. These measurements were taken within FEN1 reaction buffer in 10 mm quartz cuvettes. The bandpass was set at 1 nm and concentrations of samples were kept diluted such that the absorbance measurements were below 0.1. This measure was taken into consideration to minimize the reabsorption effect. pCy3 absorption was quantified by integrating with ± 1 nm bounds around the absorption maximum (535 nm). The instrumental baseline was recorded before each measurement with a blank sample containing FEN1 reaction buffer. For each sample, the absorbance measurements of DF substrates in the absence of FEN1 (A_i) were recorded, then FEN1 was added at a saturating concentration to the same cuvette and incubated for 3 mins at room temperature. Following that, the absorbance (A_f) of labeled DF-substrates was recorded in the presence of FEN1. Dilution due to the volume change upon FEN1 addition was accounted for when performing further calculations. Similar to the SS fluorescence change, the absorbance change upon FEN1 addition to the DF substrates was calculated as follows:

Absorbance Change = 100 * (A_i -A_f)/ A_i. The reported measurements are the average of 3 independent replicates.

O-328 Absorbance and Fluorescence emission Spectra

The absorption and fluorescence emission spectra of O-328 in the absence and presence of K^+, and in comparison with those of free Cy3 dye, free Cy3B dye, and an internally-labeled pCy3 oligo were acquired at room temperature using a microplate spectrofluorometer (TECAN infinite M1000) in water or RPA reaction buffer, respectively. Absorption spectra were collected from 440 nm to 575 nm and emission spectra were collected from 543 to 700 nm with an excitation at 535 nm. Absorption and fluorescence emission spectra were also collected for background absorption and emission using a water and an RPA buffer blanks. The background absorption and emission were then subtracted from the samples absorption and fluorescence emission spectra and the spectra were finally normalized to a maximum value of 1.

Fluorescence Quantum Yield Measurements

The fluorescence quantum yield measurements were performed following established protocols [1] based on fluorescence emission and absorption measurements. For each pCy3-labeled DF substrate, three fluorescence quantum yields were measured: that of pCy3-labeled 5' flap oligo and that of the DF substrate in the absence and presence of FEN1 all within FEN1 reaction buffer. For O-328 oligo, two fluorescence quantum yields were measured (in water and RPA buffer). For each quantum yield, the absorbance of the sample was measured (as described above), then the fluorescence emission spectrum of the same sample was collected (as described above). Samples were diluted and these measurements were reiterated 3 times keeping the absorbance in the range of 0-0.1 where the absorbance and fluorescence emission correlate linearly. Cy3B, dissolved in FEN1 buffer or RPA buffer for DF-substrate experiments or O-328 experiments, respectively, was used as a reference dye and same procedure was applied. Hence, the fluorescence quantum yield of Cy3 in each case was calculated according to this equation:

$$\phi_{Cy3} = \phi_{Cy3B} \frac{E_{Cy3}(1 - 10^{-A_{Cy3B}})n_{Cy3}^2}{E_{Cy3B}(1 - 10^{-A_{Cy3}})n_{Cy3B}^2} \quad (1)$$

where ϕ is the fluorescence quantum yield, E is the integrated fluorescence emission across the whole spectrum (550-700 nm), A is the absorbance at 535 nm, n is the refractive index of the medium. The values of the refractive indices are same for both the reference and sample. The fluorescence quantum yield of Cy3B (0.67) was used as reported [2].

Theoretical Non-Radiative Lifetime Loss Calculations

For the determination of the non-radiative loss of Cy3- and Cy3B-coupled DNA, the natural fluorescence lifetime (radiative lifetime, in the absence of non-radiative competing pathways) was calculated based on the widely used Strickler-Berg equation [3]. For a transition from a lower energy l to a higher energy level u, induced by light absorption, the radiative lifetime of the excited state (τ_{rad}) is given by:

$$\frac{1}{\tau_{rad}} = 2.88 \times 10^{-9} n^2 \langle \tilde{\nu}_f{}^{-3} \rangle_{Av}{}^{-1} (g_l/g_u) \int \epsilon(\tilde{\nu}) dln(\tilde{\nu}) \quad (2)$$

where n is the refractive index of the buffer, $\tilde{\upsilon}$ is the frequency of the transition expressed in cm^{-1}, $\epsilon(\tilde{\upsilon})$is the molar extinction coefficient spectra, and g_l and g_u are the degeneracies of the lower and upper states, respectively, with $(g_l/g_u) = 1$ for fluorescent transition. The integration and the averaging of the third negative power of the frequency of the transition are performed over the whole absorption spectra. This formula was initially used for atomic species, but it estimates well the radiative fluorescence lifetime for rigid fluorophores. In the presence of non-radiative competing pathways, the measured fluorescence lifetime will always be shorter than this theoretically estimated value, in correlation with the quantum yield. Therefore, the ratio between the measured fluorescence lifetime and theoretical radiative fluorescence lifetime serves as an estimate of the non-radiative competing pathways, including trans->cis loss (for reviews, consult [4]). In our experiments, the molar extinction coefficient spectra were determined by normalizing the absorption spectra over several dilutions and the refractive index was approximated by the refractive index of water. Thus, the non-radiative lifetime loss percentage was approximated by $d\tau = (\tau_{rad}-\tau_{exp})/\ \tau_{rad}*100$. For free Cy3 and Cy3B, the radiative fluorescence lifetime was estimated by dividing the published fluorescence lifetimes by the quantum yields of these two dyes [5].

Determination of the hyperbolic viscosity dependence of fluorescence lifetime and decoupling of rates

In general, for a fluorophore that can photoisomerize from an excited state that is capable of fluorescent de-excitation, the measured fluorescence lifetime τ of this state is given by:

$$\frac{1}{\tau} = k_r + k_{nr} + k_{iso} \tag{3}$$

where k_r is the rate of the fluorescent radiative de-excitation, k_{nr} is the rate resulting from all the non-radiative pathways except photoisomerization and k_{iso} is the photoisomerization rate. In the simplest model, the photoisomerization rate from any given state has been shown to depend on the viscosity of the environment through the dependence [6,7]:

$$k_{iso}(\eta) = D\,\eta^{-a}\exp(-E_0/RT) \tag{4}$$

where η is the dynamic viscosity, D is parameter associated with the rotational freedom of the fluorophore, E_0 is the height of the energy barrier that has to be crossed for photoisomerization, a is a constant between 0 and 1, R is the gas constant and T is the absolute temperature.

For simplicity, the value of the parameter a can be considered 1. Moreover, this parameter is not of interest for us as we do not intend to characterize the exact mechanism of photoisomerization. With this simplification, the measured fluorescence lifetime τ as a function of viscosity becomes:

$$\frac{1}{\tau(\eta)} = k_r + k_{nr} + \frac{D\exp(-E_0/RT)}{\eta} \tag{5}$$

Calculating the limit at saturating viscosity gives:

$$\frac{1}{\tau_\infty} = \lim_{\eta\to\infty}\frac{1}{\tau(\eta)} = k_r + k_{nr} \tag{6}$$

which yields the fluorescence lifetime at saturating viscosity, given that photoisomerization is completely inhibited. In certain experiments, this value was simply replaced by the lifetime of free Cy3B with the assumption that all the other de-excitation pathways except photoisomerization are similar for Cy3 and Cy3B[8].

The rate of isomerization at any viscosity can, therefore, be easily obtained by subtraction:

$$k_{iso}(\eta) = \frac{1}{\tau(\eta)} - \frac{1}{\tau_\infty} \tag{7}$$

We are particularly interested in the rate of photoisomerization from trans* in pure water, for which we determine the lifetime of a particular construct in pure water and in saturating glycerol.

Rearranging the terms in the first equation gives:

$$\tau(\eta) = \frac{1}{\frac{1}{\tau_\infty} + \frac{\mathrm{D}\exp\left(-\frac{E_0}{RT}\right)}{\eta}} = \frac{\tau_\infty * \eta}{\eta + \tau_\infty \mathrm{D}\exp\left(-\frac{E_0}{RT}\right)} \tag{8}$$

Making the notation $K_{1/2} = \tau_\infty \mathrm{D}\exp\left(-\frac{E_0}{RT}\right)$, the above equation becomes:

$$\tau(\eta) = \frac{\tau_\infty * \eta}{K_{1/2} + \eta} \tag{9}$$

This equation describes a Michaelis-Menten type hyperbola with the asymptotic limit τ_∞. The parameter $K_{1/2}$ represents the dynamic viscosity at which half of the maximum lifetime is achieved. At constant temperature, and since τ_∞ is an intrinsic constant of the fluorophore, $K_{1/2}$ depends on the rotational freedom of the fluorophore expressed by D and the height of the photoisomerization energy barrier E_0.

Steady State Fluorescence Anisotropy Measurements

Steady state fluorescence anisotropy measurements were carried out using QuantaMaster 800 spectrofluorometer (Photon Technology International Inc.) equipped with a Xenon arc lamp. The optical pathway was set to L-configuration. To reduce the collection of scattered light, a longpass filter (550 nm) was placed at the emission side. Measurements were recorded at room temperature in FEN1 reaction buffer. Samples were excited at 532 nm and emission was collected between 564 and 572 nm with 5 nm slit width for both excitation and emission. Emission was then integrated over the above interval. For all the below used indices, V denotes the vertical setting of a polarizer, while H denotes the horizontal setting of a polarizer. For any pair of indices, the first one denotes the setting of the excitation-side polarizer, while the second one denotes the setting of the emission-side polarizer. Every sample was prepared and split into two equal volumes in order to measure the I_{VV} and I_{VH} components independently with the scope of minimizing error introduced by photobleaching. For each sample, the analysis and measurements were performed as detailed below.

First, the instrument G-factor was determined following the below formula [9], and by measuring the I_{HH} and I_{HV} components for each sample.

$$G = \frac{I_{HV}}{I_{HH}} \tag{10}$$

Next, the I_{VV} and I_{VH} components were measured, and the steady state anisotropy r was determined according to the following equation [9].

$$r = \frac{I_{VV} - G * I_{VH}}{I_{VV} + 2 * G * I_{VH}} \qquad (11)$$

The fluorescence lifetime, anisotropy and rotational correlation time are related through the Perrin equation [9]:

$$r = \frac{r_0}{1 + \frac{\tau}{\varphi}} \qquad (12)$$

where r is the steady state fluorescence anisotropy measured as mentioned above, τ is the measured fluorescence lifetime, φ is the rotational correlation time and r_0 is the intrinsic anisotropy of the fluorophore in the absence of any rotation given by the angle between the absorption and emission dipoles.

In order to determine the rotational correlation time φ, we first experimentally determined r_0 by measuring the I_{VV} and I_{VH} components of each sample in FEN1 reaction buffer containing 60% glycerol. The 60% glycerol fully inhibited any rotational motion. Applying the above anisotropy formula, we obtained r_0 values of ~0.38, as previously reported for Cy3 [5]. Hence, the rotational correlation time φ was calculated as follows:

$$\varphi = \tau * \frac{r}{r_0 - r} \qquad (13)$$

Time-Resolved Fluorescence Anisotropy Measurements

Time-resolved fluorescence anisotropy measurements were carried out using QuantaMaster 800 spectrofluorometer (Photon Technology International Inc.) equipped with a Fianuim supercontinuum fiber laser source (Fianium, Southampton, U.K.) operating at 20 MHz repetition rate. Arrival time of each photon was measured with a Becker-Hickl SPC-130 time-correlated single photon counting module (Becker-Hickl GmbH, Berlin, Germany). Photons were counted using time to amplitude converter (TAC). The optical pathway was set to L-configuration. To reduce the collection of scattered light, a longpass filter (550 nm) was placed at the emission side. For all measurements, the counts were acquired for 1 min. Measurements were recorded at room temperature in FEN1 reaction buffer. Samples were excited at 532 nm and emission was collected at 565 nm with 5 nm slit width for both excitation and emission. The instrument response function (IRF) was estimated using a Ludox colloidal silica suspension dissolved in water.

Similar to steady state fluorescence anisotropy measurements, each sample was prepared and split into two equal volumes for separate measurements. All indices and pair of indices are as described above. For each sample, the analysis and measurements were performed as described below.

The instrument G-factor was determined by measuring the I_{HH} (t) and I_{HV} (t) components decays and by using tail-matching option in FluoFit software package (PicoQuant).

Next, the I_{VV} (t) and I_{VH} (t) components decays were measured for each sample and the start points of their multi-exponential decays were determined by using the IRF. I_{VV} (t) and I_{VH} (t) were then shifted accordingly on the time-axis, to ensure that these starting points are aligned at t_0=0 ns. The time-resolved anisotropy decay r (t) was determined according to [9]:

$$r(t) = \frac{I_{VV}(t) - G * I_{VH}(t)}{I_{VV}(t) + 2 * G * I_{VH}(t)} \quad (14)$$

This time-resolved anisotropy decay r(t) was then fitted to a model accounting for wobbling-in-cone [8,10,11] (fluorophore) with the cone attached to a cylinder (DNA) [12]:

$$r(t) = r_0\left((1 - A_\infty)e^{-\frac{t}{\tau_{Cone}}} + A_\infty\right)\left(\frac{1}{4}(3cos^2(\alpha) - 1)^2 + \frac{3}{4}sin^2(2\alpha)e^{-D_{Cylinder}t} + \frac{3}{4}sin^4(\alpha)e^{-4D_{Cylinder}t}\right) \quad (15)$$

where r_0 is the intrinsic anisotropy of the fluorophore given by the angle between the aborption and emission dipole moments, τ_{Cone} is the rotational relaxation time for the wobbling-in-cone, $D_{Cylinder}$ is the rotational diffusion coefficient of the cylinder around its long axis, α is the angle between the long axis of the cylinder and the rotational symmetry axis of the cone in the approximation that the angle between the dipoles is small (which is the case of Cy3 with r_0=0.38 which is close to 0.4) and A_∞ is a parameter related to the cone angle by: $A_\infty = \left[\frac{1}{2}\cos(\theta)(1 + \cos(\theta))\right]^2$

where θ is the maximum angle between the dipole moments of the fluorophore and the rotational symmetry axis of the cone achieved during the wobbling-in-cone motion.

The first decay component is associated with the local rotation of the fluorophore relative to the DNA molecule to which it is attached and is described by a fast wobbling-in-cone motion [8], while the second component is associated with the slow overall spinning motion of the complex around the DNA long axis.

In order to follow the fast component associated directly with the local fluorophore rotation relative to the DNA, we calculated the wobbling-in-cone diffusion coefficient (D_w), as described previously [11].

$$D_w = \frac{1}{\tau_{Cone}(1 - A_\infty)}\left\{\frac{-x^2(1 + x)^2[\log((1 + x)/2) + (1 - x)/2]}{2(1 - x)} + \frac{1 - x}{24}(6 + 8x - x^2 - 12x^3 - 7x^4)\right\} \quad (16)$$

where x=cos(θ).This wobbling-in-cone diffusion coefficient incorporates both the steric restriction imposed by the neighboring residues through its dependence on θ and the effect of the transient interactions and the solvent that can collectively slow down the rotation in their vicinity through its dependence on τ_{Cone}, while maintaining the ease of comparison between various conditions. It can be regarded as an overall measure of the fluorophore's local rotational freedom in a particular environment. Mathematically, D_w incorporates both the exponential factor of wobbling component as well as it its pre-exponential factor, which is related to the percentage contribution of this component.

Decoupling the Fluorescence Lifetime Rate Components

As described in the previous sections, the measured fluorescence lifetime τ of the excited state is given by:

$$\frac{1}{\tau} = k_r + k_{nr} + k_{iso} \tag{17}$$

where k_r is the rate of the fluorescent radiative de-excitation, k_{nr} is the rate resulting from all the non-radiative pathways except photoisomerization and k_{iso} is the photoisomerization rate.

Likewise, the radiative quantum yield ϕ, i.e. the relative efficiency of the radiative de-excitation, is given by:

$$\phi = \frac{k_r}{k_r + k_{nr} + k_{iso}} = \tau * k_r \tag{18}$$

Therefore, by measuring the fluorescence lifetime and the fluorescence quantum yield of a particular labeled construct, the rate of the radiative de-excitation is obtained algebraically as:

$$k_r = \frac{\phi}{\tau}$$

The isomerization rate in a particular buffer can be obtained through an increasing viscosity study on the construct of interest, as described in the previous sections. k_{iso} is then given by the following formula.

$$k_{iso}(\eta_{Buffer}) = \frac{1}{\tau(\eta_{Buffer})} - \frac{1}{\tau_\infty} \tag{19}$$

where η_{Buffer} is the dynamic viscosity of the buffer of interest, $\tau(\eta_{Buffer})$ is the fluorescence lifetime in the buffer conditions of interest and τ_∞ is the fluorescence lifetime at saturating viscosity. For most of the rate decoupling experiments, the buffer of interest was pure water. For simplicity, we remove the explicit viscosity dependence of the fluorescence lifetime in the buffer of interest, thus making the notation $\tau(\eta_{Buffer}) = \tau$, the isomerization rate then becomes:

$$k_{iso} = \frac{1}{\tau} - \frac{1}{\tau_\infty} \tag{20}$$

Once k_r and k_{iso} are determined, k_{nr} can be obtained through algebraic operations as:

$$k_{nr} = \frac{1}{\tau} - k_r - k_{iso} = \frac{1}{\tau} - \frac{\phi}{\tau} - \left(\frac{1}{\tau} - \frac{1}{\tau_\infty}\right) \tag{21}$$

After rearranging the terms, k_{nr} is given by:

$$k_{nr} = \frac{1}{\tau_\infty} - \frac{\phi}{\tau} \tag{22}$$

To summarize, once τ, ϕ and τ_∞ are experimentally measured, the de-excitation component rates can be decoupled by the following system of equalities:

$$\begin{cases} k_r = \frac{\phi}{\tau} \\ k_{nr} = \frac{1}{\tau_\infty} - \frac{\phi}{\tau} \\ k_{iso} = \frac{1}{\tau} - \frac{1}{\tau_\infty} \end{cases} \quad (23)$$

It is worthy to mention that the above system of equations holds as well for rigid fluorophores or non-rigid fluorophores that have been fully rigidified by external conditions. The rigidity can be tested for by performing an increasing viscosity study on the complex of interest. In the case where the fluorophore is fully rigidified, the fluorescence lifetime does not increase with the increase in viscosity. Mathematically, this observation is expressed as $\tau_\infty = \tau$. With this observation, in the case of rigid fluorophores such as Cy3B, the above-mentioned system of rates becomes:

$$\begin{cases} k_r = \frac{\phi}{\tau} \\ k_{nr} = \frac{1-\phi}{\tau} \\ k_{iso} = 0 \end{cases} \quad (24)$$

Signal-to-Noise Ratio Analysis of smFRET/smPIFQ Traces

The noise around a constant value, for a variable that takes only positive values, such as the photon count, can be defined as the reciprocal of signal-to-noise ratio (SNR), given by:

$$\frac{1}{SNR} = \frac{\sigma}{\mu} \quad (25)$$

where μ denotes the signal mean value and σ denotes the noise standard deviation (SD) around the mean signal value. For the smFRET/smPIFQ FEN1 cleavage traces, μ and σ were determined individually for each trace from the 10-15 frames preceding any state change. μ and σ were determined for a population N=100 traces in each case (smFRET/smPIFQ). The resulting set of inverse SNR values was analyzed for mean and standard error of the mean (SEM) according to the below equations:

$$\begin{cases} Mean\left(\frac{1}{SNR}\right) = \frac{1}{N} \sum_{Trace=1}^{N} \left(\frac{\sigma}{\mu}\right)_{Trace} \\ SEM\left(\frac{1}{SNR}\right) = \sqrt{\frac{\sum_{Trace=1}^{N} \left[\left(\frac{\sigma}{\mu}\right)_{Trace} - Mean\left(\frac{1}{SNR}\right)\right]^2}{N * (N-1)}} \end{cases} \quad (26)$$

The absolute fractional change (AFC) upon protein binding was then calculated for each of the N=100 traces as:

$$AFC = \left|\frac{\mu_F - \mu_I}{\mu_I}\right| = \left|\frac{\Delta\mu}{\mu_I}\right| \quad (27)$$

where μ_I denotes the mean of the signal before the protein binding, μ_F denotes the mean of the signal after the protein binding and | | denotes the absolute value function. Similar to the case of

the inverse signal to noise ratio, the set of absolute fractional change upon protein binding was analyzed for the mean and SEM as:

$$\begin{cases} Mean(AFC) = \frac{1}{N}\sum_{Trace=1}^{N}\left|\frac{\Delta\mu}{\mu_I}\right|_{Trace} \\ SEM(AFC) = \sqrt{\frac{\sum_{Trace=1}^{N}\left[\left|\frac{\Delta\mu}{\mu_I}\right|_{Trace} - Mean(AFC)\right]^2}{N*(N-1)}} \end{cases} \tag{28}$$

In order to determine whether the fluorescence/FRET change is significant beyond the noise level, the two datasets, i.e. $\left(\frac{1}{SNR}\right)_{Trace=\overline{1:N}}$ and $(AFC)_{Trace=\overline{1:N}}$ in both cases were first tested for normality using MATLAB implemented Kolmogorov-Smirnov test. Once the normality of the two datasets was confirmed, the two datasets were compared for statistical difference using two-sample t-test with a significance level α=0.001 using the MATLAB build-in function ttest2 with the options set to allow for unequal SDs of the two sets. The statistic of this test is given by:

$$t = \frac{Mean(AFC) - Mean\left(\frac{1}{SNR}\right)}{\sqrt{[SEM(AFC)]^2 + \left[SEM\left(\frac{1}{SNR}\right)\right]^2}} \tag{29}$$

Supplementary Figures and Legends

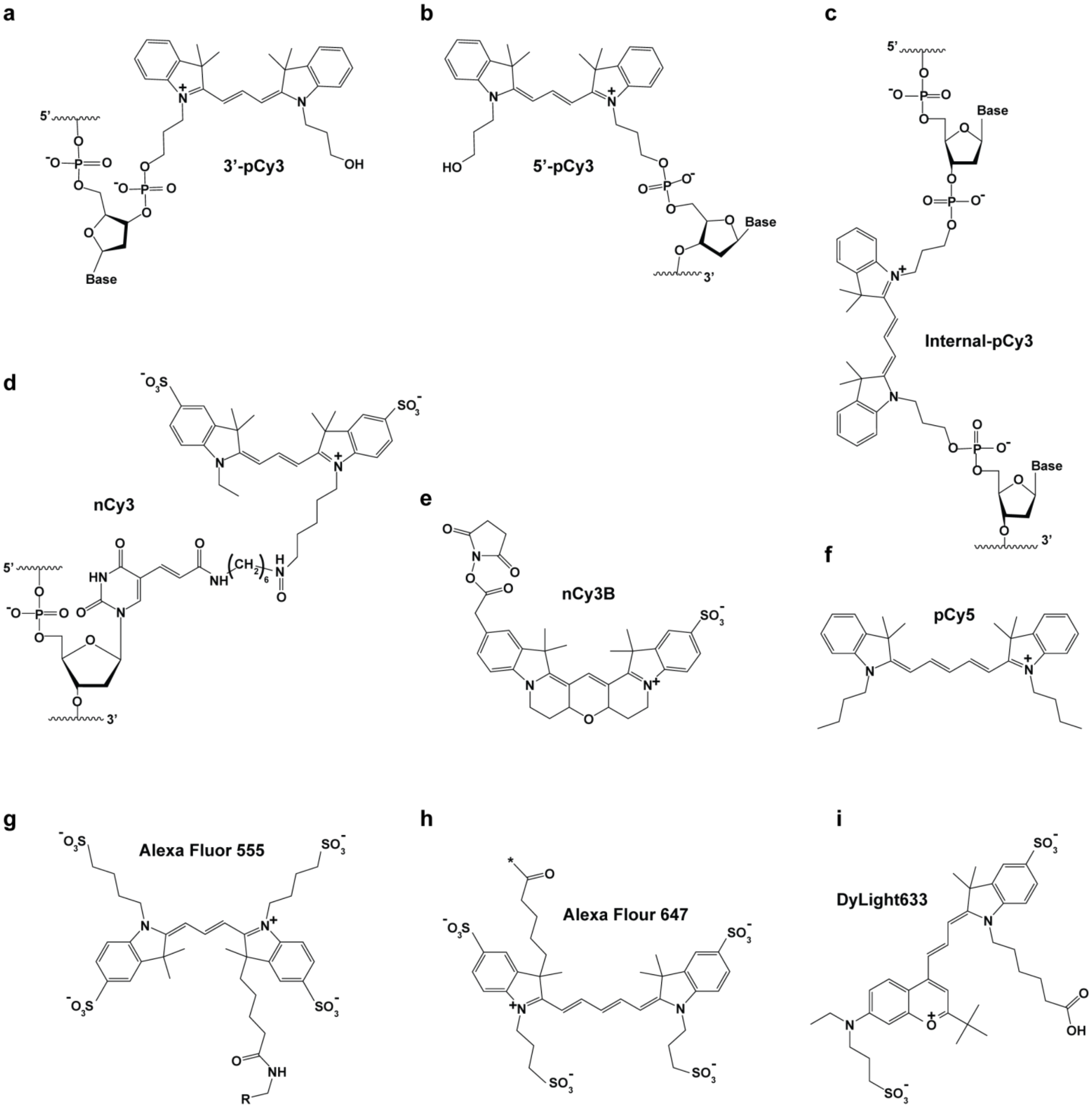

Supplementary Fig. 1. Structures of the different fluorophores used in this study: **(a)** 3'-phosphoramidite-linked-Cy3 (3'-pCy3) **(b)** 5'-pCy3 **(c)** internal pCy3 **(d)** Cy3-NHS (nCy3) **(e)** Cy3B **(f)** pCy5 **(g)** Alexa Fluor 555 **(h)** Alexa Fluor 647 and **(i)** DyLight 633.

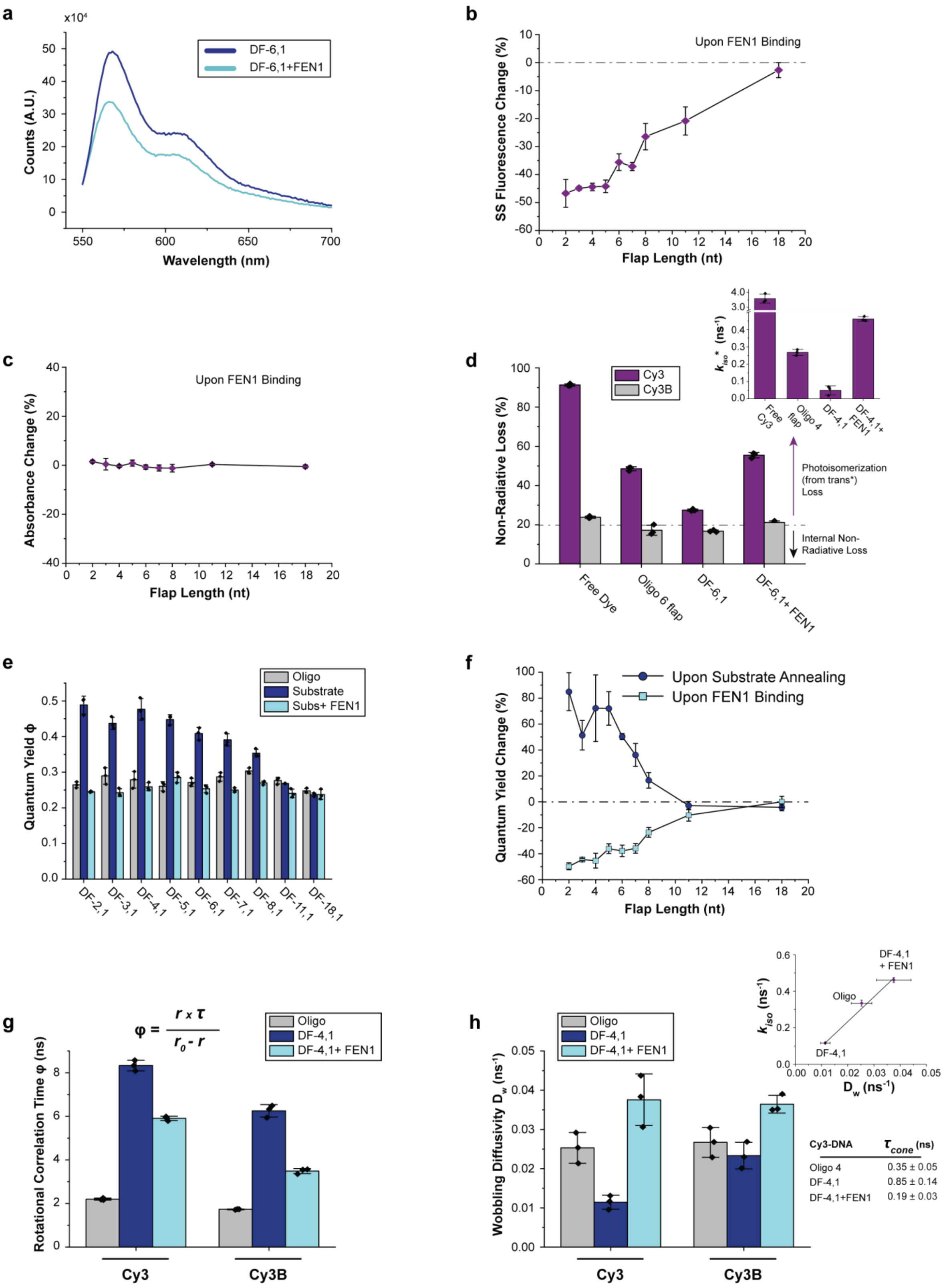

Supplementary Fig. 2. (a) Steady state emission spectra of DF-6,1 substrate, with and without FEN1 showing that the Cy3 fluorescence of the DF substrate is quenched by FEN1. **(b)** Graph showing the flap length dependence of FEN1-induced quenching of DF substrates, with flap

lengths varying from 2 to 18. This quenching is calculated from steady state emission measurements. **(c)** Graph showing percentage of change in ground state absorption of the DF substrate, with and without FEN1. **(d)** Bar chart graph showing the loss of the excited-state lifetime due to non-radiative de-excitation, including the photoisomerization from trans*. This loss is calculated, as described in SI Methods section, for Cy3 (purple) and Cy3B (grey) dyes, in the following four forms: free dye, bound to oligo, and in DF substrate with and without FEN1. The horizontal dashed line indicates the average total non-radiative loss in Cy3B for the four forms. Further loss (above the dashed line) in Cy3 is attributed to the photoisomerization loss from trans* excited state. Inset shows the photoisomerization rate (k_{iso}) of Cy3 in the above-mentioned forms. Except for the FEN1-bound DF substrate form, k_{iso} was estimated as described earlier through an increasing viscosity study. For FEN1-bound DF substrate and since the protein binding will be inhibited at saturating glycerol concentrations, τ_{∞} was approximated by that of Cy3B. The rate of photoisomerization indicates that some degree of photoisomerization is present in all three conditions and that it strongly anti-correlates with their measured fluorescence lifetimes. **(e)** Bar chart graph showing the fluorescence quantum yield of Cy3 in oligo alone, upon making the DF substrate and upon addition of FEN1 to DF substrate for flap lengths 2-18. **(f)** Graph showing percentage of change in quantum yield, upon annealing of the DF substrate and FEN1 binding to the annealed substrate as a function of flap length. **(g)** Bar chart representing the rotational correlation time φ (ns), as determined from steady state anisotropy measurements, for oligos (grey), DF-substrates (blue) and DF-substrates in the presence of FEN1 (cyan). The rotational correlation times in this 3-context system were determined for pCy3- and Cy3B-labeled DF-4,1, as described in SI Methods. The formula for calculating φ is included. The decrease in rotational correlation time upon FEN1 binding to DF-4,1 clearly points to the destabilization of the existing interactions between the dye and the dsDNA in the DF substrate context, as FEN1 binding can only lead to an increase in the gyration radius of the complex, and hence, an increase in the rotational correlation time. **(h)** Bar chart showing the wobbling-in-cone diffusion coefficient (D_w), as determined from time-resolved anisotropy measurements, for both DF-substrates in the 3-context system as described for g and SI Methods. Inset shows the correlation between k_{iso} (calculated by approximating τ_{∞} to that of Cy3B) and D_w for pCy3-labeled oligo, DF-4,1 in the absence and presence of FEN1. The data is fitted with linear regression yielding a slope of 13.57±0.96 and a Pearson coefficient of 0.99. Inset table reports τ_{cone} (ns) for the pCy3-labeled constructs. The results in g and h indicate that FEN1 binding releases a fast-rotating component that was reduced in the substrate, and that this component has a stronger impact than the overall complex spinning rate reduction. In the case of Cy3B, these interactions are reduced as compared to pCy3, probably due to the differences in the overall charge and hydrophobicity of the two dyes. Error bars and ± represent SD from 3 replicates.

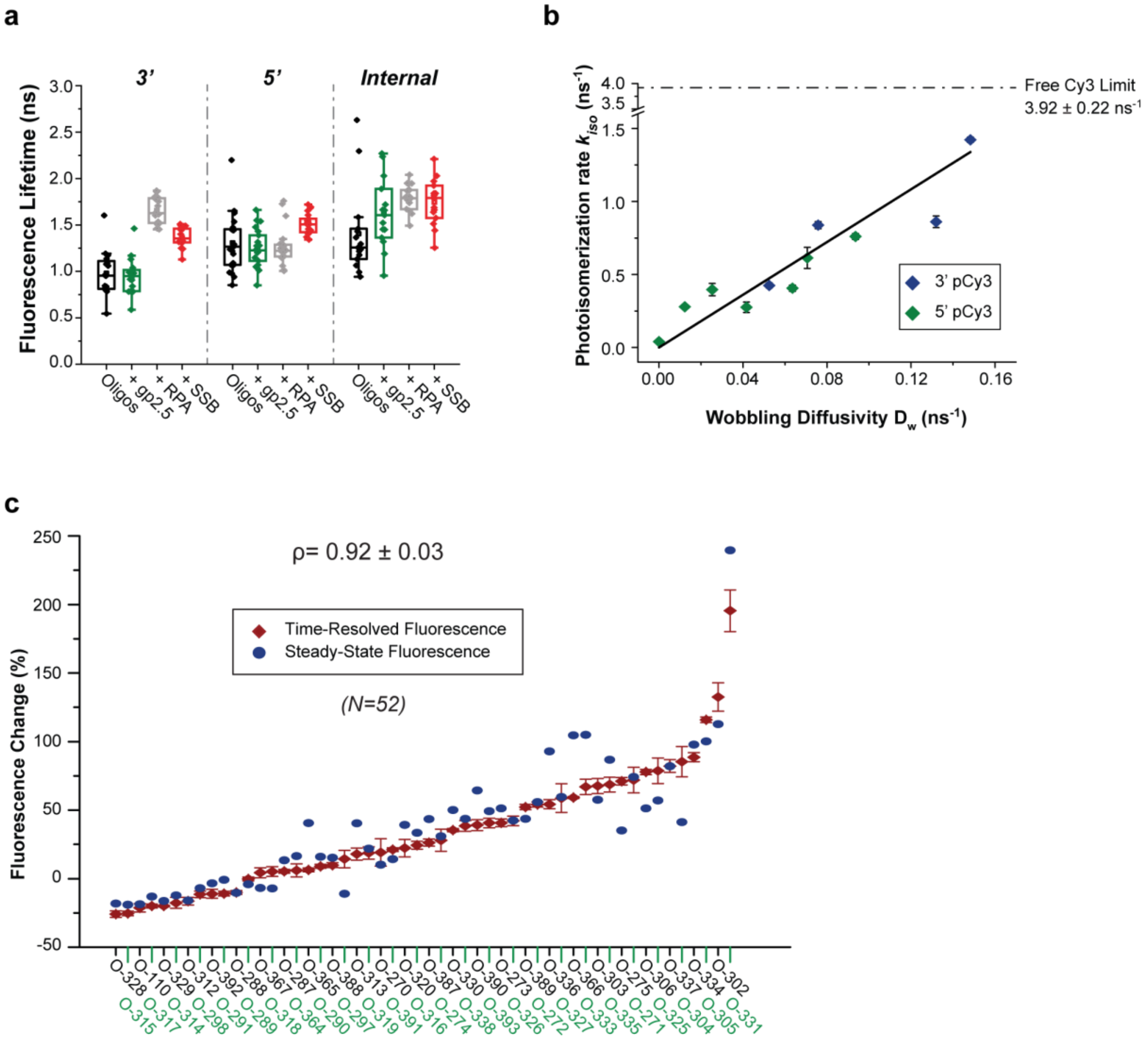

Supplementary Fig. 3. (a) Box chart summarizing the distribution of the lifetimes for the 3 libraries (3'-, internally, and 5'-labeled), in the absence (black) and presence of one of the ssDNA binding proteins; gp2.5 (green), RPA (grey) or SSB (red). For these libraries, RPA or SSB binding narrowed down the variation of the distribution of lifetimes by ~2-3 folds, as indicated by the difference in SDs of the distributions upon protein binding and the deviation in the datasets containing oligos only. On the other hand, binding of gp2.5 did not significantly affect the variation of the lifetime distributions in all three libraries. **(b)** Graph showing the correlation between k_{iso} (calculated by approximating τ_∞ to that of Cy3B) and D_w for a subset of 3'- (blue) and 5'-pCy3-labeled oligos (green). Horizontal dashed line represents the k_{iso} upper limit of free Cy3. The data is fitted with linear regression yielding a slope of 9.0±0.6 and R^2-value of 0.95. Error bars and ± represent SD of 3 replicates. **(c)** Plot showing the change in fluorescence (%) upon RPA binding to various oligos from all 3 libraries shown in Fig. 3b-d. The fluorescence change determined by time-resolved fluorescence measurements (red) and steady-state fluorescence measurements (blue) are both shown with significant agreement between the two measurements as indicated by Pearson coefficient. Error bars represent SD from 3 replicates and ± represents SEM.

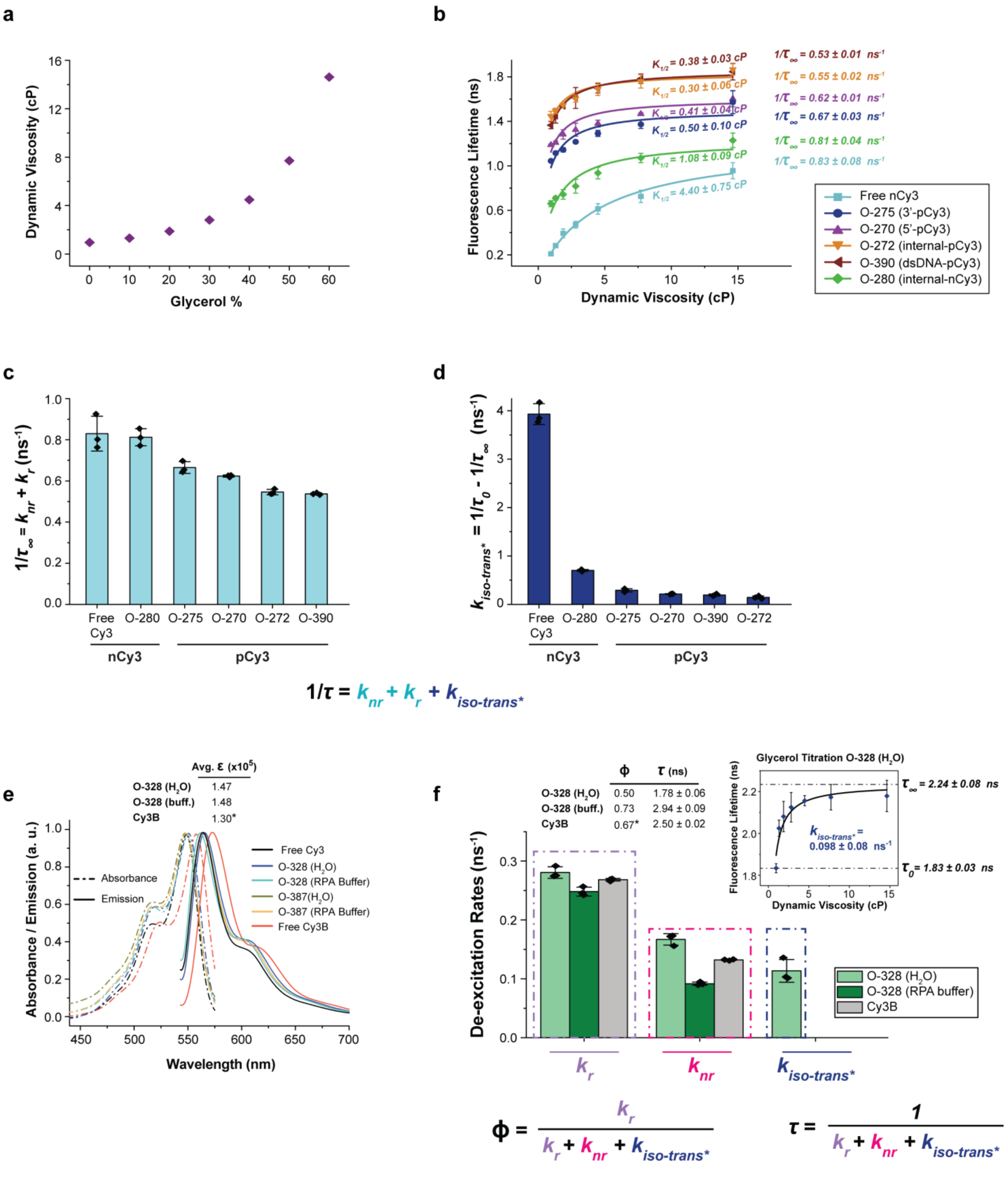

Supplementary Fig. 4. *(***a***)* Conversion curve from glycerol percentage in water to dynamic viscosity. **(b)** Dependence of the fluorescence lifetime for the different constructs, shown in Fig. 4*A*, on dynamic viscosity. Each curve was fitted to a Michaelis–Menten type hyperbola, as described in the SI Methods section. $K_{1/2}$ values and the inverse value of τ_∞ is indicated for each construct. **(c)** Bar chart of the inverse values of the lifetimes in saturating glycerol τ_∞, for each of the constructs shown in Fig. 4a. **(d)** Bar chart of the photoisomerization rates from trans* in pure

water of the constructs shown in Fig. 4a. The equation below c and d describe the rate components, photoisomerization-independent (in Cyan) and –dependent (in blue), of the measured lifetime for any fluorophore in a particular condition. **(e)** Absorbance (dashed lines) and emission (solid lines) spectra of free Cy3 dye (black), O-328 oligo measured in water (blue), O-328 oligo measured in RPA buffer (cyan), random internal-labeled oligo measured in water (green), random internal-labeled oligo measured in RPA buffer (yellow) and free Cy3B dye (red). The spectra were corrected for background absorbance or emission by subtracting the absorbance or emission, respectively, of a blank solution (water or RPA buffer). These spectra were then normalized to a maximum value 1. **(f)** Bar chart representing the decoupled de-excitation rates; radiative component (k_r) due to radiative decays, non-radiative component (k_{nr}) due to non-radiative pathways excluding photoisomerization, and photoisomerization-dependent component ($k_{iso\text{-}trans*}$), of O-328 in water (light green), O-328 in RPA buffer (green) and free Cy3B dye (grey) used as a reference. These rates were decoupled as described in SI Methods. Inset table shows the quantum yield (ϕ) and the measured fluorescence lifetime (τ) of the different samples. The quantum yield of Cy3B (0.67, as reported earlier [13]) is used as a reference to calculate the other quantum yields. Inset graph shows the fluorescence lifetime (ns) as a function of dynamic viscosity (cP), as determined from increasing viscosity study. The graph is fit with Michaelis–Menten type hyperbola as described in SI Methods, and $k_{iso\text{-}trans*}$ is reported. Error bars and ± represent SD from 3 replicates.

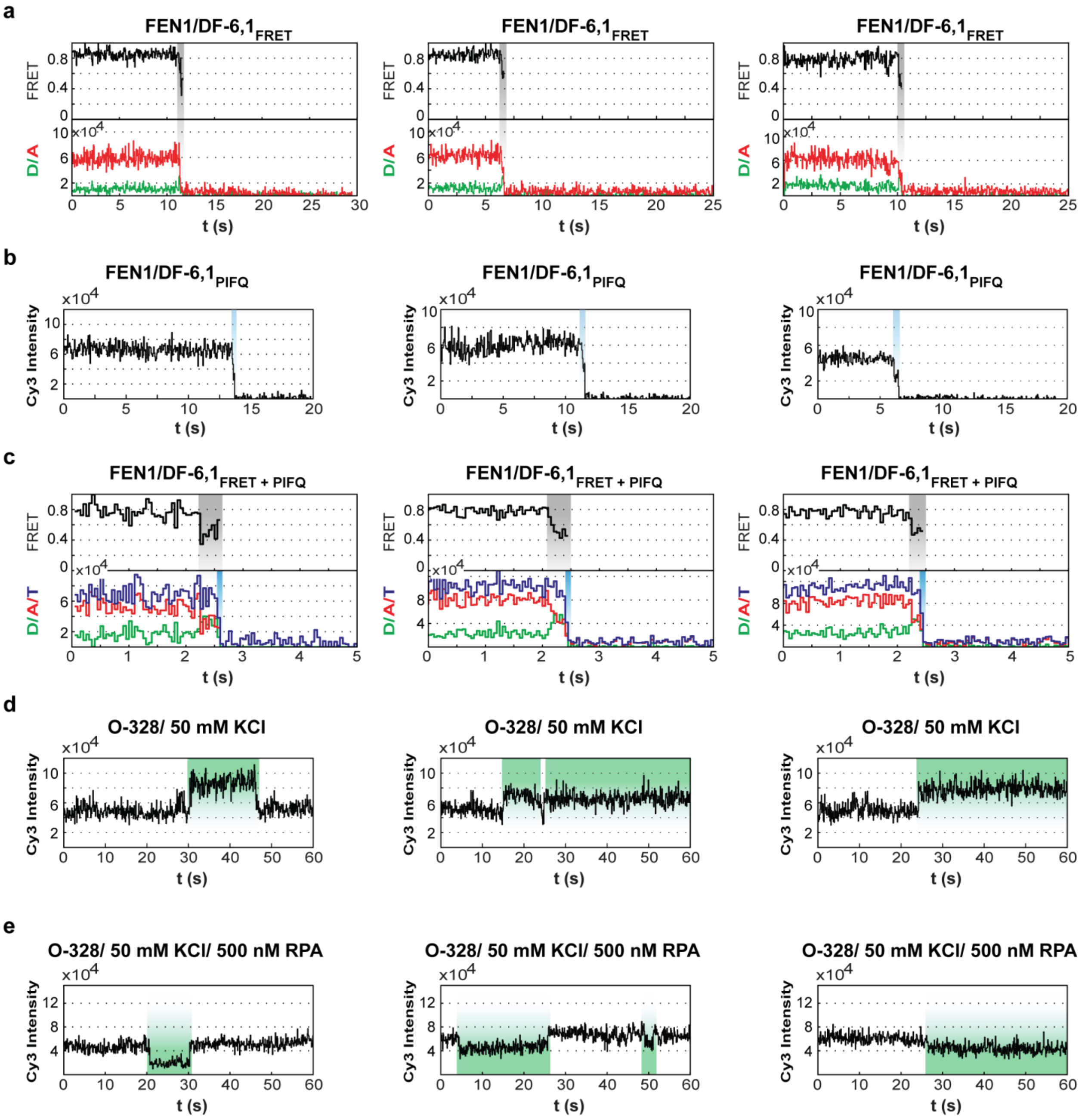

Supplementary Fig. 5. (a) Representative smFRET traces from cleavage assay of DF-6,1 by FEN1, as described in Fig. 5a. **(b)** Representative smPIFQ traces from cleavage assay of DF-6,1 by FEN1, as described in Fig. 5b. **(c)** Representative smFRET traces from cleavage assay of DF-6,1 by FEN1 monitoring both FRET change and total intensity change, as described in Fig. 5c. **(d)** Examples of time traces of the secondary structure formation, within the context of O-328 and upon addition of K^+ as well as their corresponding Cy3 fluorescence enhancements, as shown in Fig. 6a. **(e)** Additional time traces of the melting of the secondary structure by RPA, within O-328 and in the presence of K^+. Traces show the quenching of Cy3 fluorescence, upon interaction of RPA with O-328, as illustrated in Fig. 6b.

Supplementary Table 1. List of all the DNA oligos and substrates used in this study.

Name	Oligo Sequence (FEN1 system)
**DF Subs.**	**DF substrate = template + 3' Flap + 5' Flap**
Template	GATGACGAGCAGTCCTAACTGGAAATCTAGCTCTGTGGAG
3' Flap	CTCCACAGAGCTAGATTTCCC
	**5' Flap (phosphoramidite)**
Flap2	/pCy3/ (or/pCy5/) TAAGTTAGGACTGCTCGTCATC
Flap3	/pCy3/TTAAGTTAGGACTGCTCGTCATC
Flap4	/pCy3/ (or/pCy5/) TTTAAGTTAGGACTGCTCGTCATC
Flap5	/pCy3/TTTTAAGTTAGGACTGCTCGTCATC
Flap6	/pCy3/ (or/pCy5/) TTTTTAAGTTAGGACTGCTCGTCATC
Flap7	/pCy3/TTTTTTAAGTTAGGACTGCTCGTCATC
Flap8	/pCy3/TTTTTTTAAGTTAGGACTGCTCGTCATC
Flap11	/pCy3/TTTTTTTTTTAAGTTAGGACTGCTCGTCATC
Flap18	/pCy3/TTTTTTTTTTTTTTTTTAAGTTAGGACTGCTCGTCATC
	**5' Flap for NHS labeling**
Flap2-N	/5AmMC6T/AAGTTAGGACTGCTCGTCATC
Flap4-N	/5AmMC6T/TTAAGTTAGGACTGCTCGTCATC
Flap6-N	/5AmMC6T/TTTTAAGTTAGGACTGCTCGTCATC
	**Alternative 5' Flap Sequences**
Flap6-seq2	/pCy3/GAAAAAGTTAGGACTGCTCGTCATC
Flap6-seq3	/pCy3/CCCTTAGTTAGGACTGCTCGTCATC
Flap6-seq4	/pCy3/AAAAAAGTTAGGACTGCTCGTCATC
	**pCy3 incorporated within 5' Flap**
Flap5-pCy3 (4)	T/pCy3/TTTAAGTTAGGACTGCTCGTCATC
Flap6-pCy3 (4)	TT/pCy3/TTTAAGTTAGGACTGCTCGTCATC
Flap7-pCy3 (4)	TTT/pCy3/TTTAAGTTAGGACTGCTCGTCATC
Flap8-pCy3 (4)	TTTT/pCy3/TTTAAGTTAGGACTGCTCGTCATC

Single Molecule Substrates

	**DF6,1-FRET**
5' Flap	/pCy3/TTTTTAAGTTAGGACTGCTCGTCATC
3' Flap	CTCCACAG/A647-dA/GCTAGATTTCCC
Template	/biotin/GATGACGAGCAGTCCTAACTGGAAATCTAGCTCTGTGGAG
	**DF6,1-PIFQ**
5' Flap	/pCy3/TTTTTAAGTTAGGACTGCTCGTCATC
3' Flap	CTCCACAGAGCTAGATTTCCC
Template	/biotin/GATGACGAGCAGTCCTAACTGGAAATCTAGCTCTGTGGAG
	**P/T Junction**
Primer (P)	TGACCGTTGTTTGACGGTCGTG/biotin/
Template (T)	CACGACCGTCAAACAACGGTCA TTTTTTTTTTTTTTTTTTTTTTT TTTTTTTTTTTTTTT GGAGGGACGG/pCy3/GGCAGGAGGAG

Name	Oligo Sequence (RPA system)
	**3'-pCy3-labeled library**
O-275	TGAATGAATGACTGCCTGACT/pCy3/
O-302	TGAATGAATGACTGCCAAAAG/pCy3/
O-303	TGAATGAATGACTGCCAAGAG/pCy3/
O-304	TGAATGAATGACTGCCCCCCC/pCy3/
O-305	TGAATGAATGACTGCCAATAG/pCy3/
O-306	TGAATGAATGACTGCCTTCCC/pCy3/
O-330	TAAGTTAGGACTGCTCGTCAT/pCy3/
O-331	AAAAAAAAAAAAAAAAAAAAA/pCy3/
O-333	GAGAAGCCCGGTGCCGAGAAG/pCy3/
O-334	CTCTCTCTCTCTCTCTCTCTC/pCy3/
O-335	TTCTTCAGTTCAGCCATCCAT/pCy3/
O-336	AAGAAGAGTTTACTGTGAAGA/pCy3/
O-337	TTTTTTTTTTTTTTTTTTTTT/pCy3/
O-338	CCCCCCCCCCCCCCCCCCCCC/pCy3/
O-365	GGGAAGCCCGGTGCCGAAGGG/pCy3/
O-366	ACTGACTGACTGGGGGGGGGG/pCy3/
	**5'-pCy3-labeled library**
O-110	/pCy3/TAAGTTAGGACTGCTCGTCATC
O-270	/pCy3/TGAATGAATGACTGCCTGACT
O-287	/pCy3/GAAAAGAATGACTGCCTGACT
O-288	/pCy3/GAGAAGAATGACTGCCTGACT
O-289	/pCy3/TGCGCGAATGACTGCCTGACT
O-290	/pCy3/CCCCCGAATGACTGCCTGACT
O-291	/pCy3/CCCTTGAATGACTGCCTGACT
O-297	/pCy3/GAAAAAGTTAGGACTGCTCGTCATC
O-298	/pCy3/CCCTTAGTTAGGACTGCTCGTCATC
O-312	/pCy3/TAAGTTAGGACTGCTCGTCAT
O-313	/pCy3/AAAAAAAAAAAAAAAAAAAAA
O-314	/pCy3/CTCTTCAGTTCAGCCATCTCA
O-315	/pCy3/GAGAAGCCCGGTGCCGAGAAG
O-316	/pCy3/CTCTCTCTCTCTCTCTCTCTC
O-317	/pCy3/TTCTTCAGTTCAGCCATCCAT
O-318	/pCy3/AAGAAGAGTTTACTGTGAAGA
O-319	/pCy3/TTTTTTTTTTTTTTTTTTTTT
O-320	/pCy3/CCCCCCCCCCCCCCCCCCCCC
O-329	/pCy3/GGCAGGAGGAGAGGATGGAGG
O-364	/pCy3/GGGAAGCCCGGTGCCGAGAAG
O-367	/pCy3/GGGGGGGGGGACTGACTGACT

Name	Oligo Sequence (RPA system)
	**Internal-pCy3-labeled library**
O-271	TGAA/pCy3/GAATGACTGCCTGACT
O-272	TGAATGAA/pCy3/GACTGCCTGACT
O-273	TGAATGAATGAC/pCy3/GCCTGACT
O-274	TGAATGAATGACTGCC/pCy3/GACT
O-325	TTTTTTTTTTT/pCy3/TTTTTTTTTTT
O-326	CCCCCCCCCCC/pCy3/CCCCCCCCCCC
O-327	AAAAAAAAAAA/pCy3/AAAAAAAAAAA
O-328	AGGAGGGACGG/pCy3/GGCAGGAGGAG
O-387	TTGATGAGCCCA/pCy3/GGAAGTTG
O-388	GCTCTGGTTGT/pCy3/GAGCCAACG
O-389	CCCTGATAA/pCy3/AAAAGCCTACC
O-390	ACCGAAGGAACAT/pCy3/TCGTCCG
O-391	AATT/pCy3/GGGGTTGCTGTCGTAA
O-392	GGTGGTGGTGGTTG/pCy3/GGTGGT
O-393	GAAAACC/pCy3/GTACTTCCAATCC
	**Oligos for NHS labeling**
O-278-N	/5AmMC6T/TGAATGAATGACTGCCTGACT
O-279-N	TGAA/iAmMC6T/GAATGACTGCCTGACT
O-280-N	TGAATGAA/iAmMC6T/GACTGCCTGACT
O-281-N	TGAATGAATGAC/iAmMC6T/GCCTGACT
O-282-N	TGAATGAATGACTGCC/iAmMC6T/GACT
O-283-N	TGAATGAATGACTGCCTGAC /3AmMC6T/
	**pCy5 Oligos**
O-278-pCy5	/pCy5/TGAATGAATGACTGCCTGACT
O-279-pCy5	TGAA/pCy5/GAATGACTGCCTGACT
O-280-pCy5	TGAATGAA/pCy5/GACTGCCTGACT
O-281-pCy5	TGAATGAATGAC/pCy5/GCCTGACT
O-282-pCy5	TGAATGAATGACTGCC/pCy5/GACT
O-283-pCy5	TGAATGAATGACTGCCTGACT/pCy5/

Supplementary References

1 Wurth, C., Grabolle, M., Pauli, J., Spieles, M. & Resch-Genger, U. Relative and absolute determination of fluorescence quantum yields of transparent samples. *Nat Protoc* **8**, 1535-1550, doi:10.1038/nprot.2013.087 (2013).

2 Dempsey, G. T., Vaughan, J. C., Chen, K. H., Bates, M. & Zhuang, X. Evaluation of fluorophores for optimal performance in localization-based super-resolution imaging. *Nat Methods* **8**, 1027-1036, doi:10.1038/nmeth.1768 (2011).

3 Strickler, S. J. & Berg, R. A. Relationship between Absorption Intensity and Fluorescence Lifetime of Molecules. *The Journal of Chemical Physics* **37**, 814-822, doi:10.1063/1.1733166 (1962).

4 Berezin, M. Y. & Achilefu, S. Fluorescence lifetime measurements and biological imaging. *Chemical reviews* **110**, 2641-2684, doi:10.1021/cr900343z (2010).

5 Sanborn, M. E., Connolly, B. K., Gurunathan, K. & Levitus, M. Fluorescence properties and photophysics of the sulfoindocyanine Cy3 linked covalently to DNA. *The journal of physical chemistry. B* **111**, 11064-11074, doi:10.1021/jp072912u (2007).

6 Murphy, S., Sauerwein, B., Drickamer, H. G. & Schuster, G. B. Spectroscopy of cyanine dyes in fluid solution at atmospheric and high pressure: the effect of viscosity on nonradiative processes. *The Journal of Physical Chemistry* **98**, 13476-13480, doi:10.1021/j100102a008 (1994).

7 Agmon, N. & Kosloff, R. Dynamics of two-dimensional diffusional barrier crossing. *The Journal of Physical Chemistry* **91**, 1988-1996, doi:10.1021/j100291a061 (1987).

8 Steffen, F. D., Sigel, R. K. O. & Börner, R. An atomistic view on carbocyanine photophysics in the realm of RNA. *Physical Chemistry Chemical Physics* **18**, 29045-29055, doi:10.1039/C6CP04277E (2016).

9 Lakowicz, J. R. *Principles of Fluorescence Spectroscopy*. Third Edition edn, (Springer Science+Business Media, LLC, 2006).

10 Kinosita, K., Jr., Kawato, S. & Ikegami, A. A theory of fluorescence polarization decay in membranes. *Biophysical journal* **20**, 289-305, doi:10.1016/s0006-3495(77)85550-1 (1977).

11 Lipari, G. & Szabo, A. Effect of librational motion on fluorescence depolarization and nuclear magnetic resonance relaxation in macromolecules and membranes. *Biophysical journal* **30**, 489-506, doi:10.1016/S0006-3495(80)85109-5 (1980).

12 Clegg, R. M. A simple derivation of the luminescence anisotropy decay from randomly distributed cylinders rotating about a single axis. *Journal of fluorescence* **16**, 761-771, doi:10.1007/s10895-006-0133-5 (2006).

13 Cooper, M. *et al.* Cy3B: improving the performance of cyanine dyes. *Journal of fluorescence* **14**, 145-150 (2004).
